# The biological and biochemical composition of wheat (*Triticum aestivum*) as affected by the bio and organic fertilizers

**DOI:** 10.1186/s12870-023-04120-2

**Published:** 2023-02-23

**Authors:** Sobhi F. Lamlom, Ahsan Irshad, Walid F. A. Mosa

**Affiliations:** 1 Plant Production Department, Faculty of Agriculture Saba Basha, Alexandria University, Alexandria, 21531 Egypt; 2 Key Laboratory of Molecular Medicine and Biotherapy, School of Life Sciences, Beijing Institute of Technology, Beijing, China; 3 Plant Production Department (Horticulture-Pomology), Faculty of Agriculture, Alexandria University, Saba Basha, Alexandria, 21531 Egypt

**Keywords:** Arbuscular mycorrhizae, Azotobacter, Organic fertilizer, Wheat

## Abstract

Microorganisms and organic compounds (humic and fulvic acid) offer viable alternatives to insecticides and mineral fertilizers. Even though many studies have shown the effects of biofertilizers and organic substances separately, little information is available on plant responses to the combined application of these bio-stimulants, even though these biological inputs have a high potential for simultaneous action. A two-year (2020/21–2021/22) field experiment was conducted to investigate the impact of organic and biofertilizers application on the growth, yield, and biochemical attributes of wheat (cv. Misr-1). Pre-planting, wheat seeds were inoculated with two biofertilizers including Mycorrhizae, and Azotobacter, and their combination (MIX), and control (un-inoculation) were considered the main plot factor. The subplot factor contained the foliar sprays of humic acid, fulvic acid, and control (no spray). The results revealed that the seed inoculation with mycorrhizae and azotobacter in combination with foliar-applied humic acid markedly (*p* ≤ 0.05) affected the growth, yield, and seed biochemical composition of wheat. Combination of mycorrhiza and azotobacter significantly (*p* ≤ 0.05) increased) plant height (100 cm), crop growth rate (18.69 g), number of spikelets per spike (22), biological yield (13.4 ton ha-1), grain yield (5.56 ton ha-1), straw yield (8.21 ton ha-1),), nitrogen (2.07%), phosphorous (0.91%), potassium (1.64%), protein content (12.76%), starch (51.81%), and gluten content (30.90%) compared to control. Among organic fertilizers, humic acid caused the maximum increase in plant height (93 cm), crop growth rate ( 15 g day-1 m-2),1000 grain weight (51 g), biological yield ( 11ton ha-1), grain yield (4.5 ton ha-1), protein content (11%), chlorophyll content (46 SPAD), and gluten (29.45%) as compared to all other treatments. The foliar application of humic acid combined with the mycorrhizae or azotobacter seed inoculation was efficient to induce wheat vegetative growth development, as well as yield and its components.

## Introduction

Wheat is one of the most cultivated crops in the world because a third of the world's population depends on it in their food. It is characterized by the ability to grow under different environmental conditions and variant agricultural systems [1]. The global average of grain yield productivity is currently at 3.3 t ha^−1^, but this rate will need to nearly double in order to meet rising food demands [2, 3]. Egypt consumes 16 million tons of wheat per year but only produces 9 million tons, so they have set a national target of increasing wheat output to meet domestic demand via the implementation of novel agricultural practices and the use of wheat varieties with the potential to increase grain production (FAO, 2019). Intensive farming practices, which permit high yield and quality, require extensive use of chemical fertilizers, which are costly chemical fertilizers. Moreover, the application of unrenewable substance inputs causes ecological damage, such as adulteration of surface water and soil water and alteration of denitrification processes [4]. In this regard, interest in ecologically friendly, sustainable, and organic agriculture techniques has lately increased [5]. To reduce environmental pollution, it is essential to develop and use sustainable agriculture methods and biofertilization [6]. The usage of bio stimulants for plant development, whose purpose is to enhance physiological processes in plants, boost nutrient acquisition, and raise tolerance against abiotic and biotic challenges, is also required by this agroecological paradigm [7–9].

For the development of wheat and other crop species, biofertilizers are being explored as a potential substitute method to get rid of environmental residues of chemical fertilizers. These biofertilizers are primarily based on beneficial microbes added to soil or seed to increase the quantity and biological activity of desirable microorganisms in the rhizosphere, hence improving soil fertility and plant development [10]. Because soil is a complex system that can be influenced by a variety of factors [11, 12], Strengthening the beneficial microbial populations in the soil, particularly in the rhizosphere area, is essential for the circulation of both organic and inorganic nutrients. This can boost nutrient availability to plants while also enhancing soil quality [13]. Bio-fertilizers can enhance plant growth via nitrogen fixation, phytohormone, phosphate, and potassium solubilization [14]. The use of azotobacter chroococcum as biofertilizers has a promising effect on maize growth and yield when compared to non-inoculated plants, according to this study.

Arbuscular mycorrhizal fungi (AMF) are advantageous microorganisms that form a symbiotic relationship with plant roots, enhancing the uptake of essential nutrients such as phosphorus (P), nitrogen (N), sulfur (S), potassium (K), calcium (Ca), iron (Fe), copper (Cu), and zinc (Zn). Additionally, they boost the absorption of nitrogen and phosphorus, increasing crop yield [15–18]. Arbuscular mycorrhizae can improve the stomatal conductance in shoots and thereby improve the photosynthetic rate in particular under drought conditions [19]. Arbuscular mycorrhiza can increase yield. Recently, in most sustainable food production systems, the application of AMF took more interest [20–22], to improve the plant nutrient, and lessen the excessive demand for chemical fertilizers [23]. It was noticed that arbuscular mycorrhizae can improve the soil structure by raising the soil water-holding capacity [24] and supplying the plants with water and nutrients [25]. Underwater scarcity, it was noticed that treating maize with arbuscular mycorrhizae improved the stomatal conductance and the plant biomass [26]. Treating wheat with arbuscular mycorrhizal stimulates changes in the composition of the leaf amino acid, and harvest index [27].

Azotobacter bacteria can use atmospheric nitrogen in the synthesis of their cellular protein and consequently improved the plant crop [28]. Besides, azotobacter could also increase the availability of iron and its absorption [29, 30]. Besides, Azotobacter can fix atmospheric nitrogen and turn it into ammonia, which is easy to be absorbed and utilized by plants [31]. Moreover, Azotobacter could improve plant protection against root pathogens [32, 33], encouraging soil beneficial microorganisms and consequently improving crop productivity [34]. Azotobacter can fix nitrogen, and produce siderophore, polysaccharides, and indole acetic acid ( IAA) that raise plant health [35–37]. Inoculation of wheat with azotobacter increased the grain yield compared with untreated plants [38]. It was found by many authors that the usage of azotobacter might encourage o the production of plant growth hormones such as auxins and gibberellins and therefore it could ameliorate the development of the plant roots, and thus could increases nodulation, nitrogen fixation and crop productivity [39–41]. Furthermore, azotobacter can improve plant health by boosting the production of Indole-3-Acetic Acid, enhancing resistance to abiotic and biotic stress and pesticides, fixing atmospheric nitrogen, increasing soil fertility, reducing soil clumps, and mitigating soil degradation [42].

Humic and fulvic acids are organic compounds that are generated from the breakdown of organic matter, such as plants and animals [43, 44]. These acids have been found to have a significant impact on soil fertility and plant nutrition, leading to their widespread use as natural soil amendments and plant growth boosters in agriculture [45]. Humic acid can soluble easily in water, and has several potential benefits for soil microbial populations and soil structure such as improving nutrient uptake, and plant growth [46]. In corn, humic acid reduces the negative impacts of water shortage and boosts the survival of droughtstress in corn [47]. Spraying of humic acid can raise the survival of plants to drought stresses [48, 49]. Besides, it was documented by Szczerski et al. [50] and Haider et al. [51] that the application of humic acid can increase the element's absorption and usage and thus the obtained crop yielding. Moreover, the same authors added that spraying of humic materials could also boost the plant height, leaf no., shoot fresh and dry weight, total sugar, carbohydrates, amino acids, minerals, and yield. Moreover, humic acid can improve the root system development, and element absorption [52], and stimulate plant growth and development and thus the obtained yield because its effect is like to the influence of the plant growth hormones; cytokinin and auxin, and gibberellin [53]. Besides, it can also increase the absorption of iron and zinc, which were involved in the synthesis of indole acetic acid [54].

On the other hand, fulvic acid is rich in macro and microelements as well as amino acids and can raise the rate of nutrient absorption from the soil as it works as a carrier to the substances from external parts to the internal parts of the plants. Because of the low molecular weight of fulvic acid, it could pass through the pores of membranes easily [55, 56], and can stay steadily in the soil solution under high salt concentrations as well as in a broad range of pH [57], so it could stimulate the developing of lateral roots and shoots and increase the crop quality attributes [58–61]. Fulvic acid can arrange plant growth development by ameliorating the photosynthetic rate and reducing transpiration conductance [62, 63]. Moreover, it is also important to boost plant growth by raising the fertilizers utilization efficiency [44], reducing the heavy metal influence [64], and increasing the yield by improving the soil's nutritional status [65].

Considering the importance of biofertilizers and organic fertilizer in the agricultural sector, this research was conducted to investigate the individual and combined application of grains inoculation with biofertilizers; mycorrhizae, and azotobacter and the exogenous application of organic fertilizer; humic acid, and fulvic acid on the performance, productivity, yield components, and quality of wheat.

## Materials and methods

### Study area

A field experiment was organized at Hosh Isa district, El-Beheira Governorate, Egypt (27°12′16.7"N 31°09′36.9" E). wheat grains (Cv. Misr 1) were sown on the 18th and 20th of November in 2020–2021 and 2021–2022, respectively. Physicochemical analysis of the experimental soil during the study seasons was shown in Table 1. Soil samples were taken from every plot at two using a spiral auger of 2.5 cm diameter. The soil samples were dried at 40 °C and ground to a size of < 2 mm. The organic matter content and soil N content were determined through wet oxidation determination and the Kjeldhal method, respectively [66]. The phosphorus and potassium contents were determined by spectrophotometry and flame photometer, respectively.

**Table 1:** The physiochemical properties of the experimental soil

Soil Properties	Season 2020		Season 2021	
	Mean	SD	Mean	SD
Mechanical analysis:				
Clay %	17.5	0.25	20	0.36
Sand %	70	1.83	68.5	1
Silt %	12.5	1.5	11.5	0.87
Soil texture	Sandy loam			
Chemical properties				
pH (1:1)	8.03	0.02	8.12	0.06
EC (1:1, water extract (ds/m)	7.9	0.1	8.2	0.21
Organic matter content %	0.96	0.03	0.91	0.03
Calcium carbonate content%	5.7	0.26	5.3	0.21
pH (1:1)	8.03	0.04	8.12	0.01
EC (1:1, water extract (ds/m)	7.9	0.25	8.2	0.30
Soluble cations (1: 2) (cmol/kg soil)				
Ca^2+^ meq/L	18.3	0.26	17.2	0.15
Mg^2+^ meq/L	8.96	0.01	9.03	0.01
Na+ meq/L	57	1	57.39	0.8
K^+^ meq/L	2.66	0.02	2.36	0.03
Soluble anions (1: 2) (cmol/kg soil)				
HCO_3_^−^ meq/L	21.3	0.11	20.25	0.03
Cl^−^ meq/L	22.25	0.02	23.24	0.19
SO_4_^2−^ meq/L	56.6	1.43	56	1
Available nutrients				
Nitrogen (N) mg/kg	219	0.3	218.7	0.21
Phosphorus (P) mg/kg	22.9	0.12	22.7	0.2
Potassium (K) mg/kg	420	1.8	425	1

The values in the table are the mean of three replicates. *SD* standard deviation. *EC* electrical conductivity

### Treatments and experimental design layout

In this experiment, four bio-fertilizer treatments including: Control (CK), Azotobacter, Mycorrhizae, and Mycorrhizae + Azotobacter (MIX), were randomly assigned to the main plots, where the organic fertilizer, humic acid (Humic Acid 70% powder, humate (Tianjin) International Limited, Tianjin, China), fulvic acid (Fulvic Acid-100% Water Soluble Fulvic Acid Powder Organic Fertilizer Hebei, China), and control, were allocated to the subplot in a split-plot design with three replicates. The soil was prepared by two orthogonal plowings, followed by leveling the soil and dividing it into the experimental plots (4 × 3 m). Nitrogen fertilizer in the form of urea (46%N) at the rate of 100 kg urea/hectare (50% of the recommended dose) was applied in two equal doses in which the first dose was applied before the first irrigation, whereas the second dose was applied before the second irrigation. During the soil preparation, 200 kg/hectare (or 50% of the advised dose) of calcium superphosphate (15.5 percent P2O5) was applied. Along with the initial dosage of nitrogen fertilizer, 50 kg/hectare (or 50% of the necessary dose) of potassium fertilizer in the form of potassium sulfate (48 percent K_2_O) was administered. Wheat grains were inoculated before sowing with Azotobacter chroococcum bacteria (Biogen), conc.106 cells/mL. Biogein is produced by the Bio-fertilizers Unit, General Organization of Agriculture Equalization Fund, Agricultural Research Centre, Giza, Egypt. Using a mixture of Glomus mosseae, Glomus fusciulatum, and Glomus clarum, the mycorrhizae, or arbuscular mycorrhizae fungi, were replicated in pot cultures with onion and maize cultivated for four months in a 1:1:1 ratio (v:v:v) Vermiculite is perlite.: peat according to Badr El-Din et al. [67]. The growing medium, spores, hyphae, and roughly cut root pieces made up the mycorrhizae inoculums. Mycorrhizae were acquired from the Plant Pathology Research Institute, Agricultural Research Center, Ministry of Agriculture, and Land Reclamation. Prior to planting, wheat grains were coated with each of the product’s mycorrhizae and Azotobacter using a sticking agent (5 percent Arabic gum). Two regimens of organic fertilizer 4% humic acid and 4% fulvic acid were utilized in this study. Additionally, two applications of foliar fertilizers were made: once at the start of spikes and again 30 days later.

### Data recording

#### Plant samples

The first sampling for estimation of crop growth was made 40 days after sowing (DAS). Subsequently, the sampling was made at 14 days intervals. To record the growth parameters, plants from an area of one square meter were harvested at ground level. The fresh weight of the whole sample was recorded, and plants were divided into their component fractions (leaves, stem, and spikes when they appeared) and weighed in fresh status. A subsample of 10 g from each fraction was taken and dried in the electrical oven maintaining a constant temperature of ± 70 °C to get constant weight. Crop growth rate (CGR) was determined based on seasonal growth data using the formulae suggested by [68] and opted by Rafiq et al. [69], and values are shown in g m^−2^ d^−1^:

1 $$\mathrm{CGR}=\left({\mathrm{Wt}}_2-{\mathrm{Wt}}_1\right)\;/\;\left({\mathrm T}_2-{\mathrm T}_1\right)$$

Wt_1_ and Wt_2_ are the total dry weights of samples (g m^−2^) at the first and second sampling, and T_2_ and T_1_ are the duration (days) between the two sampling dates.

Chlorophyll content (SPAD) measurements were taken from the base, the middle, and the tip of the flag leaf on each tiller three times during each growing season [70]. Chlorophyll concentration was calculated by averaging SPAD data (Konica Minolta Optics Inc., Tokyo, Japan). The height (in cm) of 10 plants from the ground up in each plot were measured at harvest. The lengths of ten randomly selected spikes were measured (in centimeters). By counting the amount of fertile and sterile spikelets on 10 randomly selected spikes, the NSS was computed. The term "1000 grain weight" refers to the weight of 1000 grains (TGW, g). After being harvested, each plot's worth of grain was packed, threshed, and measured in tons per hectare (GY). Straw yield (SY) was calculated by weighing the straw that was collected from a given subplot after threshing and then converting that weight to tons per hectare. Before threshing, plants were collected from a designated area within each subplot, and their total weight was recorded as the biological yield (BY), expressed as tons per hectare (ton ha ^1^). The Harvest index (HI) was calculated according to the following formula

2 $$\mathrm{HI}=\frac{\mathrm{Grain}\;\mathrm{yield}}{\mathrm{Biological}\;\mathrm{yield}}\;\times\;100$$

#### Qualitative analysis

Various quality characteristics were determined by the methods described by the American Association of Cereal Chemists [71] and [72]. Grain samples were taken from each treatment, and N, P, and K percentages were determined, grains were dried then, they were crushed and stored for further analysis. A 0.5 g of the grains powder was wet-digested with an H_2_SO_4_–H_2_O_2_ mixture. Using Nessler's approach, total nitrogen in digested plant matter was calculated calorimetrically. The measurement was taken at 420 nm, and N was calculated using the following formula:

3 $$\mathrm N\;\%=\;{\mathrm{NH}}_4\%\;\times\;0.78$$

The protein content in the wheat grains was calculated using the Formula as follows:

4 $$\mathrm{Total}\;\mathrm{protein}=\mathrm N\;\mathrm{content}\;(\%)\;\times\;6.25$$

Using a JENWAY 6305 UV/Visible Spectrophotometer at 643 nm (OD643) and the colorimeter technique, grain phosphorus was measured (ammonium molybdate). Using a flame photometer (BWB Model BWB-XP, 5 Channel), the potassium content of seeds was assessed by Motsara and Roy [73].

Starch contents were determined by Omeg Analyzer G, where an 18-mm sample spacer was used to fill wheat grains in a machine sample hopper and digital reading of starch content was noted from the screen display, according to the procedure [72]. Gluten values of grains were estimated by glutomatic 2200 apparatus, by using sodium chloride solution [74].

### Data analysis

The general linear model (GLM) algorithm of the SAS 9.4 program for Windows was used to perform the analysis of variance (ANOVA) for all analyzed features [75]. The data were examined at a 0.05 level using Fisher's least significant difference (LSD) test. To show the variety of foliar fertilizer and biofertilizer applications, boxplots were established. The ggplot2 package was used to build boxplots in the R project (version 3.4.5). The relationships between characteristics relating to growth, yield, and biochemical content of kernels were explored using Pearson correlation coefficients.

## Results

### Response of wheat to individual applications of fertilization

#### Studied traits performance under biofertilizer inoculation

**Fig. 1:**
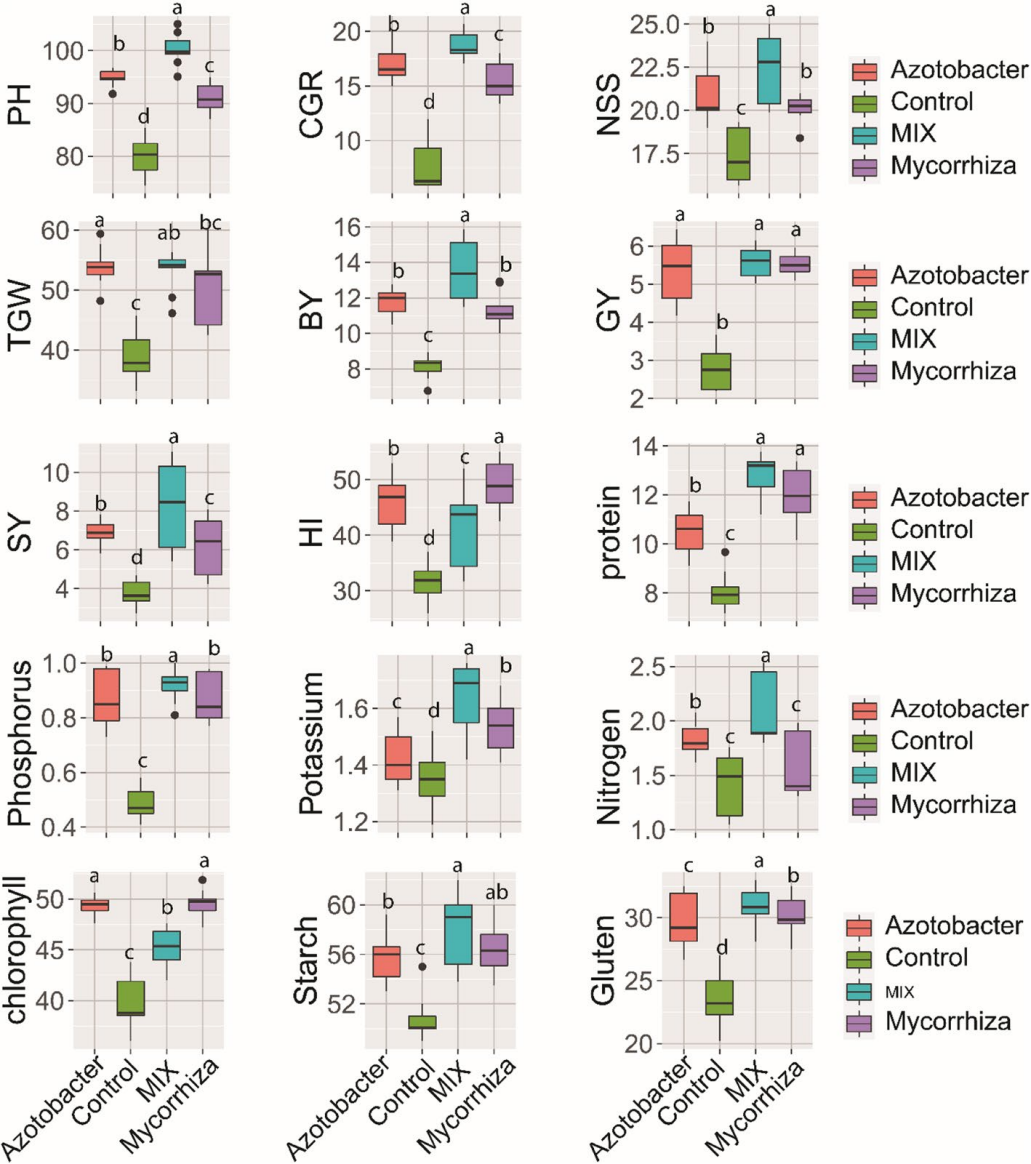
Effects of biofertilizer treatments (control, mycorrhizae, azotobacter, and mix) based on information from the seasons of 2020 and 2021 coupled with findings from 15 wheat analyzed qualities identified at field tests. Different lowercase letters on error bars indicate statistically significant differences between treatments (*p* ≤ 0.05), as performed by the least significant difference (Fisher’s LSD) test. plant height (PH), crop growth rate(CGR), number of spikelets per spike (NSS),1000 grain weight (TGW), biological yield (BY), grain yield (GY), straw yield (SY), harvest index (HI), chlorophyll content (CH), Nitrogen(N), phosphorous (P), potassium (K), protein content (PC), starch, and gluten content

Analysis of variance (ANOVA) for the effect of biofertilizer was shown in Table 2. The results showed that the application of biofertilizer affected highly significantly (*p* ≤ 0.001) plant height (PH), crop growth rate(GCR), number of spikelets per spike (NSS),1000 grain weight (TGW), biological yield (BY), grain yield (GY), straw yield (SY), harvest index (HI), chlorophyll content (CHL), phosphorous (P), potassium (K), protein content (PC), starch, and gluten content during two experimental seasons. The performance of biofertilizers on yield, growth, and seed biochemical traits is presented in Fig. 1. The application of mycorrhizae combined with azotobacter (mix) recorded the highest value of plant height (100 cm), crop growth rate (18.69 g), number of spikelets per spike (22), biological yield (13.4 ton ha^−1^), grain yield (5.56 ton ha^−1^), straw yield (8.21 ton ha^−1^),), nitrogen (2.07%), phosphorous (0.91%), potassium (1.64%), protein content 12.76%), starch (51.81%), and gluten content (30.90%). The results also showed that the differences between the application of mycorrhizae and azotobacter on chlorophyll content was so slight not enough to be significant, whereas there was no significant difference in grain yield revealed between biofertilizer treatments. Moreover, the obtained results also cleared that the harvest index was greatly increased by the addition of mycorrhizae, whereas the weight of 1000 grain was remarkably enhanced by the application of azotobacter during studying seasons.

**Table 2:** ANOVA of the effects of biofertilizers, organic fertilizer applications, and their interaction on growth, physiological, yield, and biochemical parameters of wheat plants

Yield Components										
Source of Variance	Plant height		Crop growth rate		No. of spikelets /spike		Biological yield		Grain yield	
	2020	2021	2020	2021	2020	2021	2020	2021	2020	2021
Biofertilizer (BF)	***	**	***	***	***	***	***	***	***	***
Organic fertilizer (OF)	***	***	***	***	***	***	***	***	***	***
BF*OF	*	*	***	***	***	***	***	*	***	***
CV	2.35	2.69	4.28	4.31	5.67	5.63	4.03	4.16	5.49	5.39
R^2^	0.96	0.97	0.99	0.99	0.88	0.89	0.97	0.98	0.97	0.98
RMSE	2.15	2.18	0.63	1.64	1.15	1.18	0.45	0.47	0.26	0.26
Growth attributes										
Source of Variance	1000 grain weight		Chlorophyll		Straw yield		Harvest index		starch	
	2020	2021	2020	2021	2020	2021	2021	2021	2021	2021
Biofertilizer (BF)	***	***	***	***	***	***	***	***	***	***
Organic fertilizer(OF)	***	***	***	***	***	***	***	***	***	***
BF* 0F	***	***	***	***	***	***	***	***	***	***
CV	7.39	7.41	2.25	2.24	5.48	5.45	7.44	7.42	3.11	3.13
R^2^	0.84	0.84	0.97	0.97	0.98	0.98	0.91	0.91	0.83	0.83
RMSE	3.78	3.83	1.03	1.04	0.34	0.34	3.18	3.20	1.73	1.75
Biochemical parameters										
Source of Variance	Nitrogen		Phosphorous		Potassium		Protein content		Gluten	
	2020	2021	2020	2021	2020	2021	2021	2021	2021	2021
Biofertilizer (BF)	***	***	***	***	***	***	**	**	***	***
Organic fertilizer( OF)	***	***	***	***	***	***	*	*	***	***
BF* OF	***	***	***	***	***	***	Ns	Ns	***	***
CV	2.74	3.29	3.34	5.82	3.06	3.81	8.91	8.91	4.75	4.74
R^2^	0.99	0.97	0.98	0.97	0.96	0.95	0.91	0.91	0.96	0.96
RMSE	0.04	0.053	0.03	0.05	0.045	0.051	3.18	3.20	1.20	1.21

*CV* coefficient of variation (%), *RMSE* root mean square error. *R*^*2*^ correlation coefficient

ns, *, **, *** indicate not significant, significant at 5% (*p* ≤ 0.05), 1% (p ≤ 0.01) and significant at 0.1% ( *p* ≤ 0.001) probability level, respectively

#### Effect of organic fertilizer on different studied traits

The results in Table 2 indicated that the application of organic fertilizer (humic and fulvic acid) increased significantly (*p* ≤ 0.001) plant height (PH), crop growth rate(GCR), number of spikelets per spike (NSS),1000 grain weight (TGW), biological yield (BY), grain yield (GY), straw yield (SY), harvest index (HI), chlorophyll content (CHL), phosphorous (P), potassium (K), protein content (PC), starch, and gluten content in the two seasons.

**Fig. 2:**
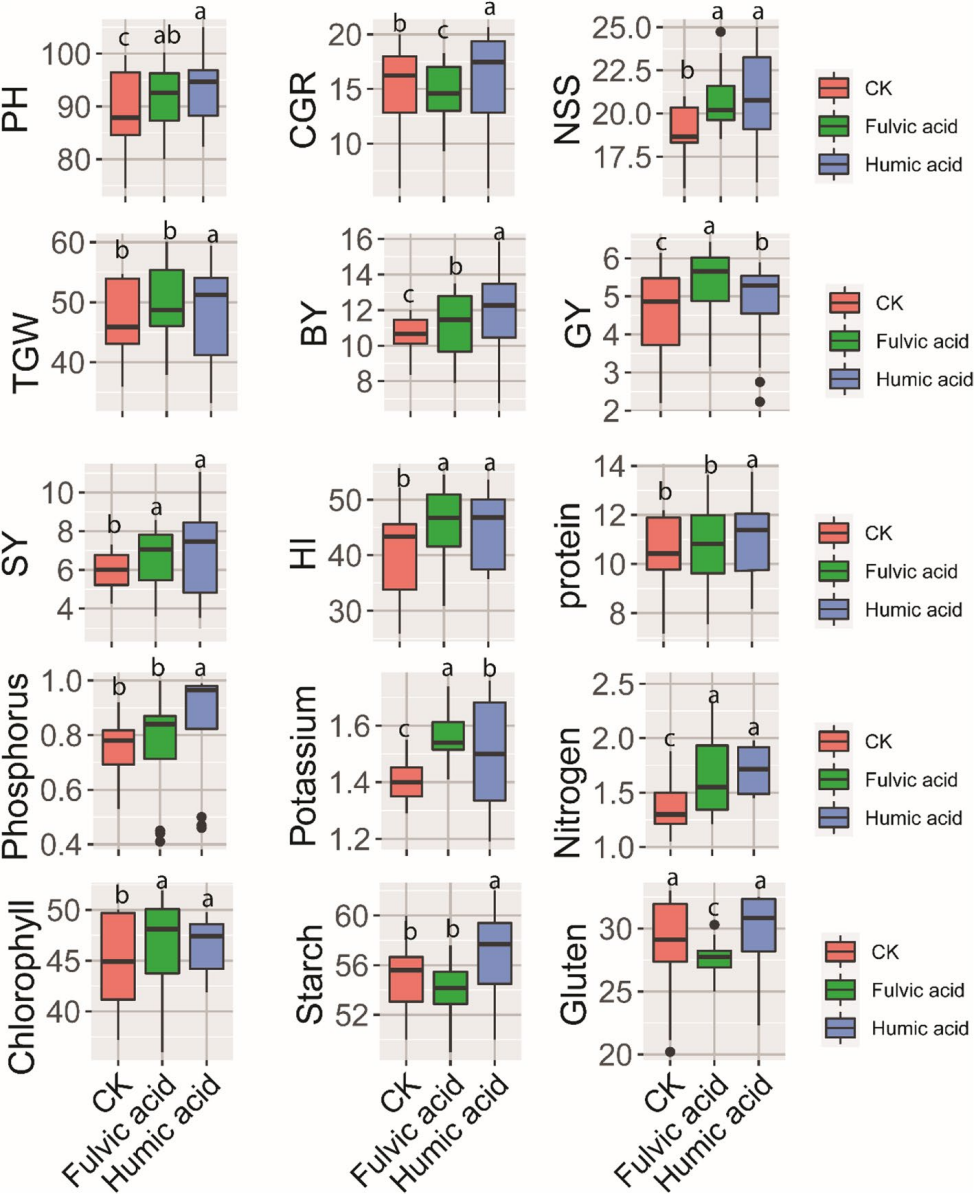
Effects of organic fertilizer treatments (control, Humic, and fulvic) on 15 studied traits for wheat were determined at field experiments. Combined analysis of 2 successive seasons of 20/21 and 2021/22. Different lowercase letters on error bars indicate statistically significant differences between treatments (*p* ≤ 0.05), as performed by the least significant difference (Fisher’s LSD) test. plant height (PH), crop growth rate(CGR), number of spikelets per spike (NSS),1000 grain weight (TGW), biological yield (BY), grain yield (GY), straw yield (SY), harvest index (HI), chlorophyll content (CH), Nitrogen(N), phosphorous (P), potassium (K), protein content (PC), starch, and gluten content

The results exhibited in Fig. 2 showed that the application of humic acid increased markedly PH (93.5 cm), CGR ( 15.19 g day^−1^ m^−2^, respectively), TGW (51.12 g,), BY( 11.80 ton ha^−1^), GY (5.21 ton ha^−1^), SY (6.53 ton ha^−1^), HI (46.11%), protein content (11.8%), K (1.56%), P (0.84%), N (1.71%), chlorophyll content (46.48 SPAD), starch (56.72%), and gluten (29.45%) compared to control and the influence of humic was higher than that of fulvic acid.

### Response of wheat to the interaction between biofertilizers and organic fertilizer treatments

#### Yield and yield component traits

The combined application of bio and organic fertilizers increased greatly the yield and yield components during the two experimental seasons (Table 2). The response of yield and yield components traits to the interaction between biofertilizers and organic fertilizer is shown in Table 3. From the results in Table 3, it could be noticed that the application of humic acid in combination with biofertilizer (Mix) recorded the highest NSS (24.05, and 24.30) in the first and second seasons, respectively over control which exhibited (15.95) in the 2020/21 season, while application of fulvic indicated the lowest NSS (16.10) in the 2021/22 season.

**Table 3:** Effects of interaction between biofertilizers and organic fertilizer treatments on wheat yield and yield components traits across two successive seasons (2020/21 and 2021/22)

Biofertilizers	Organic fertilizer	Biological yield		Grain yield		Straw yield		Harvest index		1000 grain weight		N0. of spikelets /spike	
		20/21	21/22	20/21	21/22	20/21	21/22	20/21	21/22	20/21	21/22	20/21	21/22
Control	CK	7.37f	7.40f	2.70d	2.76de	3.03 h	3.06 g	36.59 cd	36.96 cd	35.65c	36.00c	15.95e	17.63de
	Humic	8.30f	8.40f	3.35d	3.40d	3.86gh	3.93 g	40.47bcd	40.90bcd	48.43ab	48.93ab	18.93cde	19.13cde
	fulvic	8.58f	8.66f	2.25e	2.26e	4.46 g	4.46f	26.25e	26.53e	54.28ab	55.06ab	17.44de	16.10e
Mycorrhiza	CK	11.12cde	11.23cde	5.46b	5.53b	6.35def	6.40de	49.22ab	49.70ab	50.19sb	50.70ab	20.75abcd	20.96abcd
	Humic	12.44bc	12.56bc	5.70ab	5.76ab	4.43 g	4.50f	45.98abc	46.46abc	44.30bc	44.73bc	19.60bcd	19.08bcd
	fulvic	10.45e	10.53e	5.43b	5.46b	7.77bc	7.86bc	52.02a	52.56a	55.28ab	55.86ab	20.20bcd	20.40bcd
Azotobacter	CK	12.35bc	12.46bc	5.14bc	5.20bc	6.25ef	6.33de	50.77a	40.90bcd	56.30a	56.86a	21.25abc	21.43abc
	Humic	11.94bcd	12.06bcd	5.45b	5.50b	7.38bc	7.46bc	45.77abc	46.23abc	53.80ab	54.36ab	22.33abc	22.53abc
	fulvic	10.90de	11.00de	4.93c	4.43c	7.01 cd	7.10 cd	40.29bcd	40.70bcd	51.61ab	52.13ab	19.35 cde	19.56cde
Mix	CK	11.77cde	11.86cde	5.74ab	5.80ab	5.78f	5.86e	48.79ab	44.26abc	52.51ab	53.03ab	22.91ab	23.13ab
	Humic	15.48a	15.63a	6.27a	6.33a	10.61a	10.73a	33.26 cd	33.60de	58.71a	59.30a	24.05a	24.30a
	fulvic	13.23b	13.36bc	5.80ab	5.86ab	8.25b	8.36b	43.82abc	44.26abc	52.25ab	52.76ab	20.20bcd	20.40bcd

According to the least significant difference (Fisher's LSD), test, different lowercase letters indicate statistically significant differences between treatments (p 0.05)

The application of humic acid in combination with biofertilizer (mix) enhanced markedly BY (15.48, and 15.63 ton ha^−1^), SY (10.61 and 10.73 ton ha^−1^), and TGW (58.71 and 59.30 g) in the two seasons of study, respectively. In contrast, the lowest values of BY (7.37 and 7.40ton ha^−1^), SY (3.03, and 3.06 ton ha^−1^), and TGW (36.96 and 35.65 g) were obtained by control treatment in the two seasons. For GY, the highest value (6.27, and 6.33 ton/ha) was obtained when the azotobacter inoculation along with control in both seasons. The application of fulvic acid combined with mycorrhizae gave the highest value for HI (52.02, and 52.56%) as compared to the application of fulvic acid, which gave the lowest values (26.25 and 26.53%) when it was applied individually in both seasons.

#### Growth parameters

The combined application of biofertilization and organic fertilizer positively influenced growth parameters (Table 4). The highest value was recorded by the application of humic with a combination of in both seasons. The combined application of humic acid and biofertilizer significantly increased PH (105 and 102 cm) and CGR (24.05, and 24.83), while the lowest plant height(76.19, and 76.22 cm) was observed by control and fulvic acid treatment in both study seasons. Besides, the control treatment gave the lowest values from CGR. Moreover, chlorophyll content was markedly enhanced by the inoculation of wheat seeds by mycorrhizae combined with fulvic acid (50.82 and 51.33) in the two study seasons.

**Table 4:** Effects of interaction between biofertilizers and organic fertilizer treatments on wheat growth parameters across two successive seasons (2020/21 and 2021/22)

organic fertilizer Biofertilizers	Organic fertilizer	Plant height		Crop Growth Rate		Chlorophyll Content	
		20/21	21/22	20/21	21/22	20/21	21/22
Control	CK	80.84ef	76.22e	15.95e	17.29 fg	37.89e	38.30e
	Humic	83.60de	82.80de	17.44de	18.51efg	42.73d	43.13d
	fulvic	76.19f	81.11e	18.93cde	17.02 g	38.12e	38.50e
Mycorrhiza	CK	91.09c	93.46bc	20.75abcd	21.06bcde	49.55ab	49.10abc
	Humic	92.47bc	93.38bc	19.60bcd	20.06cdefg	48.62abc	49.10abc
	fulvic	89.88 cd	83.02de	20.20bcd	20.48cdef	50.82a	51.33a
Azotobacter	CK	93.60bc	91.60c	21.25abc	22.70abcd	49.47ab	50.00ab
	Humic	95.01bc	94.31bc	22.33abc	23.21abc	48.53abc	49.03abc
	fulvic	95.76bc	90.37 cd	19.35cde	20.48 cdef	49.55a	50.43a
Mix	CK	98.34ab	100.87ab	22.91ab	24.82a	42.73d	43.13d
	Humic	102.94a	105.13a	24.05a	24.83a	40.05c	46.53c
	fulvic	98.96ab	91.88c	20.20bcd	19.51defg	46.61abc	46.53c

Different lowercase letters indicate statistically significant differences between treatments (*p* ≤ 0.05), as performed by the least significant difference (Fisher’s LSD) test

#### Biochemical composition (NPK, protein, Starch, and Gluten)

The combined application of biofertilization and organic fertilizer had a highly significant effect (*p* ≤ 0.01) on all seed biochemical traits in both seasons of study (Table 2).

The difference in the performance of wheat grains’ biochemical composition under various regimes of biofertilizers combined with different organic fertilizers is shown in Table 5. The application of biofertilizer and humic acid increased statistically nitrogen content (2.09, and 2.11%), grain starch (60.33 and 60.93), and potassium content (1.73 and 1.76%) in both seasons of the study. Regarding phosphorus content, it was remarkably enhanced by the application of mycorrhiza with humic acid exhibiting the highest level of P (0.98 and 1%) in both seasons, followed by the individual application of mycorrhiza. For gluten, fulvic acid with MIX treatment exhibited the highest values of (31.99, and 32.33) while the lowest values were observed by control treatment in the two seasons. protein content was not influenced significantly by the interaction of bio-organic fertilization in both growing seasons.

**Table 5:** Effects of interaction between biofertilization and organic fertilizer treatments on wheat grains biochemical traits across two successive seasons (2020/21 and 2021/22)

Biofertilizers	Organic fertilizer	Potassium		phosphorous		nitrogen		Starch		Gluten	
		20/21	21/22	20/21	21/22	20/21	21/22	20/21	21/22	20/21	21/22
Control	CK	1.35def	1.46 cd	0.43 g	0.43d	1.70c	1.73d	50.00d	50.50d	21.00 g	21.20c
	Humic	1.23f	1.23e	0.46gf	0.50d	1.49d	1.53d	52.33 cd	52.86 cd	21.54c	21.76c
	fulvic	1.33ef	1.36de	0.55f	0.53d	1.09f	1.10 g	55.11a-d	55.67a-d	21.21c	21.43c
Mycorrhiza	CK	1.80a	1.83a	0.83de	0.83bc	2.08a	2.10a	55.21abcd	55.76a-d	30.35ab	30.63ab
	Humic	1.35def	13.36de	0.98a	1.0a	1.94b	1.96b	57.63ab	58.23ab	21.64c	28.63b
	fulvic	1.55b	1.56bc	0.86 cd	0.90abc	1.39e	1.43de	55.80abc	56.36abc	28.34b	28.63b
Azotobacter	CK	1.35def	1.36de	0.76e	0.76c	1.26e	1.30ef	55.80abc	56.40abc	30.49ab	30.80ab
	Humic	1.38cde	1.40bc	0.87bcd	0.90abc	1.48d	1.50d	56.63abc	57.16abc	21.55c	21.76c
	fulvic	1.53bc	1.56bc	0.86d	0.86abc	1.26e	1.26f	54.97bcd	55.53bcd	21.36c	21.60c
Mix	CK	1.53bc	1.56bc	0.86d	0.86abc	1.26e	1.26f	54.97bcd	55.53bcd	21.36c	21.60c
	Humic	1.73a	1.76a	0.94ab	0.97ab	2.09a	2.11a	60.33a	60.93a	21.40c	21.60c
	fulvic	1.49cde	1.50bc	0.85d	0.86abc	1.84bc	1.86bc	54.43bcd	54.96b-d	31.99ab	32.33a

Different lowercase letters indicate statistically significant differences between treatments (*p* ≤ 0.05), as performed by the least significant difference (Fisher’s LSD) test

### Correlation between studied traits

**Fig. 3:**
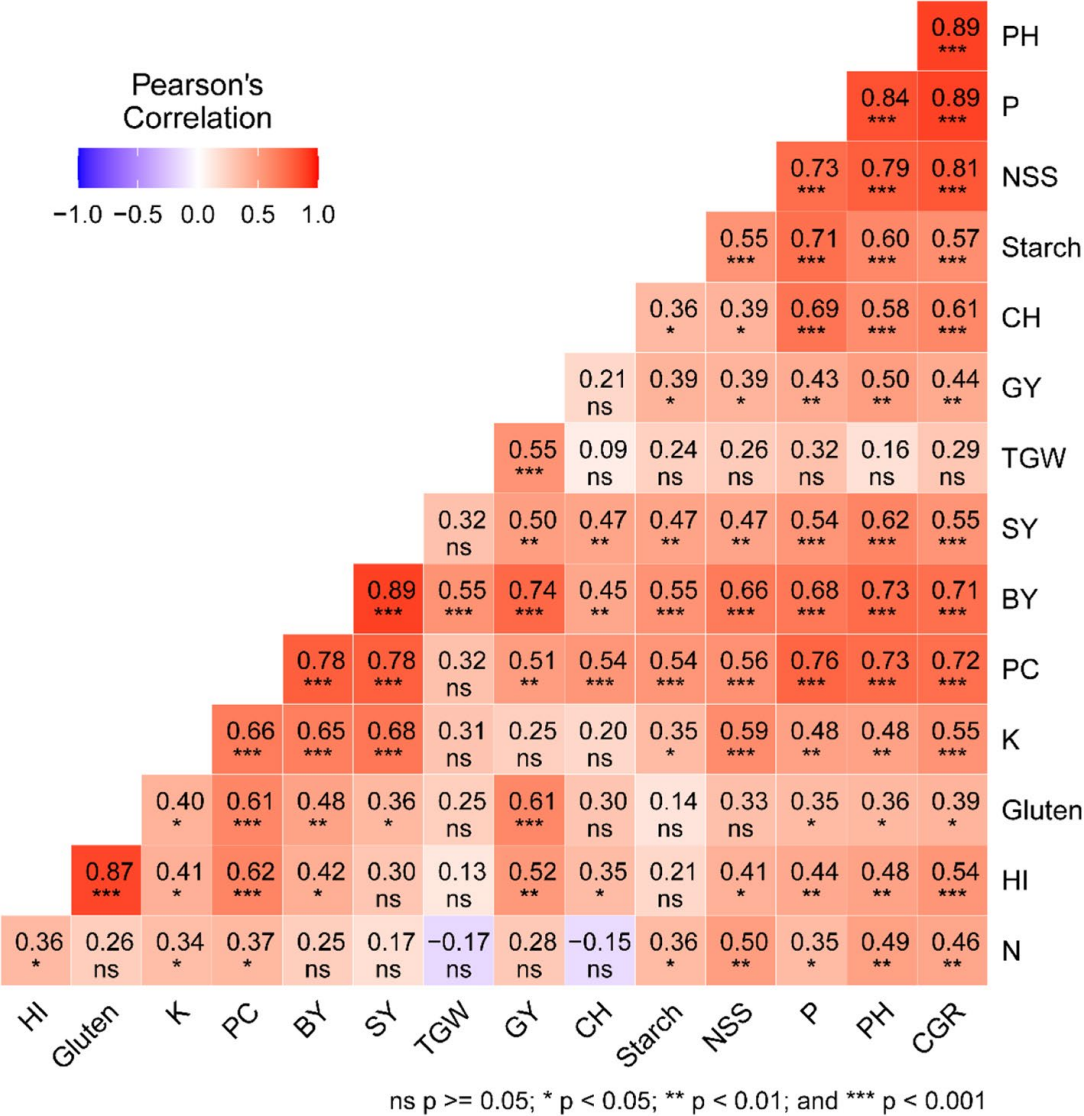
Pearson's correlation coefficients among 15 studied traits under different bio-fertilizers and organic fertilizers(Combined analysis of 2 successive seasons of 20/21 and 2021/22). plant height (PH), crop growth rate(GCR), number of spikelets per spike (NSS),1000 grain weight (TGW), biological yield (BY), grain yield (GY), straw yield (SY), harvest index (HI), chlorophyll content (CHL), nitrogen (N), phosphorous (P), potassium (K), protein content (PC), starch, and gluten

Correlation analysis among all 15 examined attributes showed strong positive correlations (Fig. 3). Amongst the yield trait pairs, the correlation between SY and BY (0.55), TGW and GY(0.55), SY and NSS (0.47), BY and NSS(0.66) were greatest, while the least correlation was observed between BY and NSS(0.39). Also, correlations among the seed biochemical traits were significantly positive, whereas other pairs of traits showed non-significant correlations, including N with each of TGW, GY, chlorophyll, Gluten, and SY (Fig. 3). Among the growth traits, the correlation between PH and CHL (0.58), followed by CGR and PH (0.89), and CHL and CGR (0.61) showed the highest significant positive coefficients (Fig. 3). As regards the correlation among TGW shows a non-significant correlation with other traits except grain yield and biological yield. As regards the correlation among different types of traits, including yield, growth, and seed biochemical parameters, highly significant positive correlations were exhibited between P and NSS (0.79), K and NSS (0.59), N and NSS (0.50), Gluten and Hi (0.87), PC and HI (0.62) (Fig. 3). protein content has a highly significant correlation with SY (0.78), and BY (0.78).

## Discussion

From the obtained results, it was clear that the inoculation of wheat plants with mycorrhiza or azotobacter individually or in combination significantly enhanced the total chlorophyll, dry weight, and crop growth rate of wheat plants (Fig. 1). These results are in the same trend as the previous findings of [76–79], they reported that mycorrhizae could assist in the intake of nutrients and consequently the yield. Moreover, mycorrhizae improved the plant's capacity in absorbing minerals; N, Ca, Mg, Fe, Cu, and Mn under salinity stress [80]. Furthermore, there was a relatively significant increase in the plant height in the tested plants [81], due to the inoculation of mycorrhiza fungi, they demonstrated higher overall morphological characteristics in soybean plants. In addition, the wheat plants significantly outperformed the control plants in terms of the number of spikelets per spike, 100-grain weight, biological yield, and grain yield. In addition, a high wheat yield in plants treated with a mixture of biofertilizers may be caused by a greater moisture content, which helps to boost the nutrient supply to plants and, as a result, raises the total yield [82]. Besides, the inoculation with Arbuscular mycorrhizae helps relieve the undesirable impacts of salinity on wheat, as well as lowers the sodium uptake, whereas it raised the availability of nitrogen, phosphorous, potassium, and magnesium and stimulated the photosynthesis process, chlorophyll, carbohydrates, and protein and thus the productivity [83, 84]. Also, it can help plants to survive drought [85] and can increase stomatal conductance, cellular and plant growth [86], and raise water uptake [87]. The association of AMF with the plant could increase the soil exploration capacity and nutritional status by increasing the absorption of potassium and reducing the Na^+^/K^+^ ratio and avoiding damaging the soil biological system [88]. Mycorrhizae raise the plant nutrient intake, and soil fertility relieves the side effect of salinity, minimizes the chemical inputs, and helps the plants to overcome the water shortage, and phytotoxic elements [89].

Azotobacter species are largely related to the composition of numerous hormones such as gibberellin, auxin, and cytokinin [90]. It could raise the wheat germination rate from 20 to 30% and associate with the absorption of nitrogen, phosphorous, iron, and zinc [91]. Azotobacter chroococcum could encourage wheat growth development and its element absorption [92]. Azotobacter spp. could induce growth and crop productivity by activating the synthesis of biological materials, encouraging the rhizosphere microbes, producing phytopathogenic controllers, and increasing the elements absorption and nitrogen fixation [93]. Azotobacter bacteria might fix about 20 kg N a year, so it could assist in ameliorating crop production [94, 95] and can minimize the demand for nitrogen fertilizers up to fifty percent [96]. Inculcation of the root with Azotobacter chroococcum increased the root system and the production of indole acetic acid [97]. Besides, inoculation of strawberries with Azotobacter spp. induced the leaf total chlorophyll content [98], improved plant nutrition, and amelioration soil fertility [99, 100].

According to the obtained results in the current study, it was obvious that humic acid application improved the growth performance, yield, and yield components of wheat. These results were previously confirmed by the findings of Muscolo et al. [90] they reported that humic acid can raise the elements absorption efficacy, and gas exchange rate in the soil, as well as can arrange the rate of stomata conductance and photosynthesis process in the plants. Moreover, humic substances have a positive impact on plant nutrition by improving N, P, mg, and Ca uptake, and thus consequently it increases the yield [101]. Besides, humic acid contains numerous nutrients that assist in improving soil fertility [102–105]. As humic acid can change positively the soil composition and its physical characteristics, so it can ameliorate plant growth and productivity by raising the chelation and availability of numerous nutrients [106–110]. It was noticed by many authors that humic acid increased beneficial microbes in the soil [111], and improved the efficiency of the used fertilizers and soil airing, so it can help in developing plant growth [112]. Potassium humate can affect positively on developing the growth, productivity, and fruit chemical composition of wheat [113]. Merwad [114] documented that humic acid can increase the absorption of NPK in wheat under salinity stress.

Fulvic acid can attract water and facilitate the mobility of nutrients like Ca, Mg, Fe, Cu, and Zn to the plants roots [45, 55]. As fulvic acid assists the transferring the elements into the plant cell, chlorophyll content, photosynthesis process rate, and minimizing the stomatal conductance and the transpiration conductance, it is considered a plant growth regulator [62, 63], and its effect is like to the influence of cytokinin, auxin and gerbilline [53, 115, 116]. Besides, it also helps in chelating mineral nutrients and increases their absorption and photosynthesis process [117–119], increasing antioxidants, gibberellic acid, cytokines and vitamins, therefore it progress the plant growth development [119–121]. Priya et al. [122] reported that applying fulvic acid can raise the intake of K and therefore it can improve the starch metabolism.

A considerable increase in plant height, crop growth rate, 100-grain weight, grain NPK content, gluten, starch, and protein were discovered in this study when mycorrhiza and azotobacter were combined with humic acid (Tables 3, 4 and 5). This result might perhaps be explained by the fact that mycorrhiza stimulates plant growth and the absorption of various crucial nutrients, such as nitrogen and phosphorus, under challenging environments. Mycorrhiza's widespread distribution throughout the coating system is responsible for this growth promotion [58]. A prior study found that the greatest harvest index was obtained when organic and biofertilizers were applied together, but there were no statistically significant differences between nitrogen applied as a biofertilizer and nitrogen applied as a chemical fertilizer [123]. When organic and biofertilizers are used in conjunction with fennel, the harvest index of fennel is lowered when compared to the control [124], which is not consistent with the findings of the current research.

## Conclusions

The current study showed that bio and organic fertilizers significantly impact the growth, yield, and grain biochemical composition of wheat plants. The best results were seen with a combination of biofertilizers and humic acid, with increased plant height, growth rate, yield, and biochemical composition. This improvement can be attributed to the better plant nutrition and nutrient efficiency provided by organic fertilizers and their synergistic effects with biofertilizers. Using humic acid in conjunction with azotobacter and mycorrhiza is a promising approach for improving wheat yields and quality.

## Data Availability

The data can be made available upon reasonable request from the Corresponding author.
